# Metformin protects from oxaliplatin induced peripheral neuropathy in rats

**DOI:** 10.1016/j.ynpai.2020.100048

**Published:** 2020-05-22

**Authors:** N.W. Martinez, A. Sánchez, P. Diaz, R. Broekhuizen, J. Godoy, S. Mondaca, A. Catenaccio, P. Macanas, B. Nervi, M. Calvo, F.A. Court

**Affiliations:** a Center for Integrative Biology, Faculty of Sciences, Universidad Mayor de Chile, Santiago 8580745, Chile; b Department of Physiology, Faculty of Biology, Pontificia Universidad Católica de Chile, Santiago, Chile; c Department of Hematology and Oncology, Pontificia Universidad Católica de Chile, Santiago, Chile; d Department of Neurology, Pontificia Universidad Católica de Chile, Santiago, Chile; e Millennium Institute on Immunology and Immunotherapy, Pontificia Universidad Católica de Chile, Santiago, Chile; f FONDAP Center for Geroscience, Brain Health and Metabolism, Santiago 8580745, Chile; g Buck Institute for Research on Ageing, Novato, San Francisco, CA 94945, USA

**Keywords:** Oxaliplatin, Metformin, Peripheral neuropathy

## Abstract

Oxaliplatin is a commonly used drug to treat cancer, extending the rate of disease-free survival by 20% in colorectal cancer. However, oxaliplatin induces a disabling form of neuropathy resulting in more than 60% of patients having to reduce or discontinue oxaliplatin, negatively impacting their chance of survival. Oxaliplatin-induced neuropathies are accompanied by degeneration of sensory fibers in the epidermis and hyperexcitability of sensory neurons. These morphological and functional changes have been associated with sensory symptoms such as dysesthesia, paresthesia and mechanical and cold allodynia. Various strategies have been proposed to prevent or treat oxaliplatin-induced neuropathies without success. The anti-diabetic drug metformin has been recently shown to exert neuroprotection in other chemotherapy-induced neuropathies, so here we aimed to test if metformin can prevent the development of oxaliplatin-induced neuropathy in a rat model of this condition. Animals treated with oxaliplatin developed significant intraepidermal fiber degeneration, a mild gliosis in the spinal cord, and mechanical and cold hyperalgesia. The concomitant use of metformin prevented degeneration of intraepidermal fibers, gliosis, and the altered sensitivity. Our evidence further supports metformin as a new approach to prevent oxaliplatin-induced neuropathy with a potential important clinical impact.

## Highlights

• After oxaliplatin treatment rats developed mechanical and cold hyperalgesia.
• We observed intraepidermal nerve fiber degeneration, and mild spinal cord gliosis.
• Co treatment with Metformin could prevent all these pathological outcomes.
• This suggests metformin as a candidate drug to prevent oxaliplatin-induced neuropathy.

## 1 Introduction

Oxaliplatin, a platinum derivative, is commonly indicated for the treatment of solid tumors such as colorectal cancer, the third most common cancer in men and the second in women worldwide (The Lancet 2018). In this disease, oxaliplatin has shown clinically important benefits in the adjuvant setting and for metastatic disease (de Gramont et al., 2000, Andre et al., 2015). However, its clinical use is associated with oxaliplatin-induced peripheral neuropathy (OIPN) which in some cases can be persistent and disabling. The incidence of chronic OIPN is between 64 and 97% with at least 12% of patients experiencing severe neuropathy. Clinical data indicates that more than 60% of patients reduce or discontinue oxaliplatin due to this side effect, impacting their chances of survival (Gebremedhn et al., 2018). OIPN is still unpredictable, while symptoms may resolve after chemotherapy is discontinued, they can also continue for years. It is associated with sensory and motor axon loss that can be detected by electrophysiology, or by reduction in intraepidermal fibre density in a length dependent pattern (Burakgazi et al., 2011). The pathophysiology of OIPN is poorly understood. In a rat model of OIPN (2 mg/kg every other day for four injections) it has been shown a reduction in intraepidermal innervation and mechanical hyperalgesia (Boyette-Davis and Dougherty 2011). Mice models of OIPN confirmed these observations (Coriat et al., 2014). Recently, a transcriptomic analysis of dorsal root ganglia (DRG) from mice treated with oxaliplatin showed that neuronal genes were the most differentially expressed by the treatment (Starobova et al., 2019). Oxaliplatin is known to form nuclear and mitochondrial DNA adducts in DRG neurons (Meijer et al., 1999, Ta et al., 2006), leading to apoptosis (Staff et al., 2017), axonal degeneration and focal demyelination. Mitochondrial damage has been described in other conditions leading to peripheral neuropathies, such as diabetes and HIV (Flatters, 2015). Interestingly, chemotherapy-induced degeneration of axons and their terminals *in vitro* is associated to intra-axonal calcium increase, mitochondrial dysfunction and elevation in ROS, and this degenerative process can be prevented by drugs that reduce mitochondrial dysfunction and ROS production (Barrientos et al., 2011, Villegas et al., 2014, Kline et al., 2019).

Currently, there are no medications recommended for the prevention or treatment of chemotherapy-induced peripheral neuropathy (Colvin 2019). Meta-analyses of clinical trials for prevention of chemotherapy-induced peripheral neuropathies report inconclusive results (Park et al., 2013, Albers et al., 2014). Treatment alternatives for established chemotherapy-induced peripheral neuropathies are also limited. Clinical trials of antiepileptic or antidepressant agents to treat other neuropathic pain conditions have generally been negative (Hammack et al., 2002, Mitchell et al., 2006, Rao et al., 2008, Rao et al., 2007). Only one double-blinded, randomized controlled trial showed some improvement in chemotherapy-induced peripheral neuropathy symptoms by duloxetine treatment after a short follow up of 5 weeks (Smith et al., 2013).

Metformin, an activator of adenosine monophosphate-activated protein kinase (AMPK), is the one of the most prescribed medications for improving glycaemic control in diabetic patients. This drug has shown neuroprotective effects in murine models of neurodegenerative disorders (Correia et al., 2008, El-Mir et al., 2008), and to decrease neuropathic pain induced by spinal nerve ligation and spared nerve injury in rats and mice, respectively (Inyang et al., 2019, Weng et al., 2019). In addition, metformin treatment attenuate hyperalgesia and allodynia in a rat model of diabetic neuropathy (Ma et al., 2015). Furthermore, in a mice model of cisplatin-induced neuropathy, metformin greatly reduced the loss of tactile sensitivity and the development of mechanical hypersensitivity (Mao-Ying et al., 2014).

Here, by using a rat model of OIPN we demonstrated that metformin strongly protects against oxaliplatin-induced degeneration of intraepidermal fibers. Furthermore, metformin is able to prevent mechanical and cold hypersensitivity produced by the chemotherapeutic treatment. Together, the data presented here open novel avenues for developing preventive therapies for OIPN in human patients.

## 2 Results

### 2.1 Metformin protects from oxaliplatin-induced axonal degeneration of sensory neurons *in vitro*

**Fig. 1:**
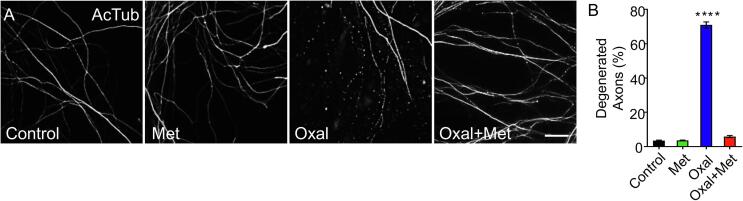
Metformin delays oxaliplatin-induced neurite degeneration *in vitro.* (A) Embryonic dorsal root ganglia explants were treated with oxaliplatin alone (10 μM), metformin alone (10 mM), or both together for 3 days. Immunostaining against acetylated tubulin (AcTub) was used to reveal the neurite cytoskeleton. Scale bar, 20 μm. (B) Percentage of degenerated neurites was determined by estimating the ratio between areas of degenerated neurites by total neurite area. Mean ± SEM, n = 3 different cultures, 5 wells per condition per culture, ****p < 0, 0001, One-Way ANOVA, Bonferroni’s post-hoc test.

Degeneration of intraepidermal nerve fibers has been suggested to be associated with sensory impairment and neuropathic pain after chemotherapy (Grone et al., 2014, Javed et al., 2014, Kosmidis et al., 2014, Martinez et al., 2015, Isak et al., 2017), therefore we wanted to first establish if metformin was able to protect oxaliplatin-induced axonal degeneration in an *in vitro* model. To this end, sensory neurons from rat embryos were cultured for 7 days and then treated with oxaliplatin (10 μM) with or without metformin (10 mM) for three days. Immunofluorescent staining against the cytoskeleton component acetylated tubulin (AcTub) was used to assess axonal morphology (Fig. 1A). Treatment of sensory neurons with oxaliplatin induces a significant degeneration of axons (Fig. 1A and B). Notably, metformin (10 mM) strongly inhibited oxaliplatin-induced axonal degeneration (Fig. 1A and B).

### 2.2 Oxaliplatin produces a distal ‘dying back’ peripheral sensory neuropathy, that can be prevented by the use of metformin

To induce OIPN *in vivo,* we treated rats with oxaliplatin (4 mg/kg i.p.) in two consecutive days per week for 4 weeks. A group of rats were concomitantly treated with metformin (250 mg/Kg daily i.p.), and a group was treated with metformin alone to serve as a control (250 mg/Kg daily i.p.). In this model of OIPN, blood sugar concentration, white blood cell counting, and animal weight were not affected in any of the groups studied (Supp. Fig. 1).

### 2.3 Oxaliplatin induces intraepidermal fibre loss, that is prevented by metformin

**Fig. 2:**
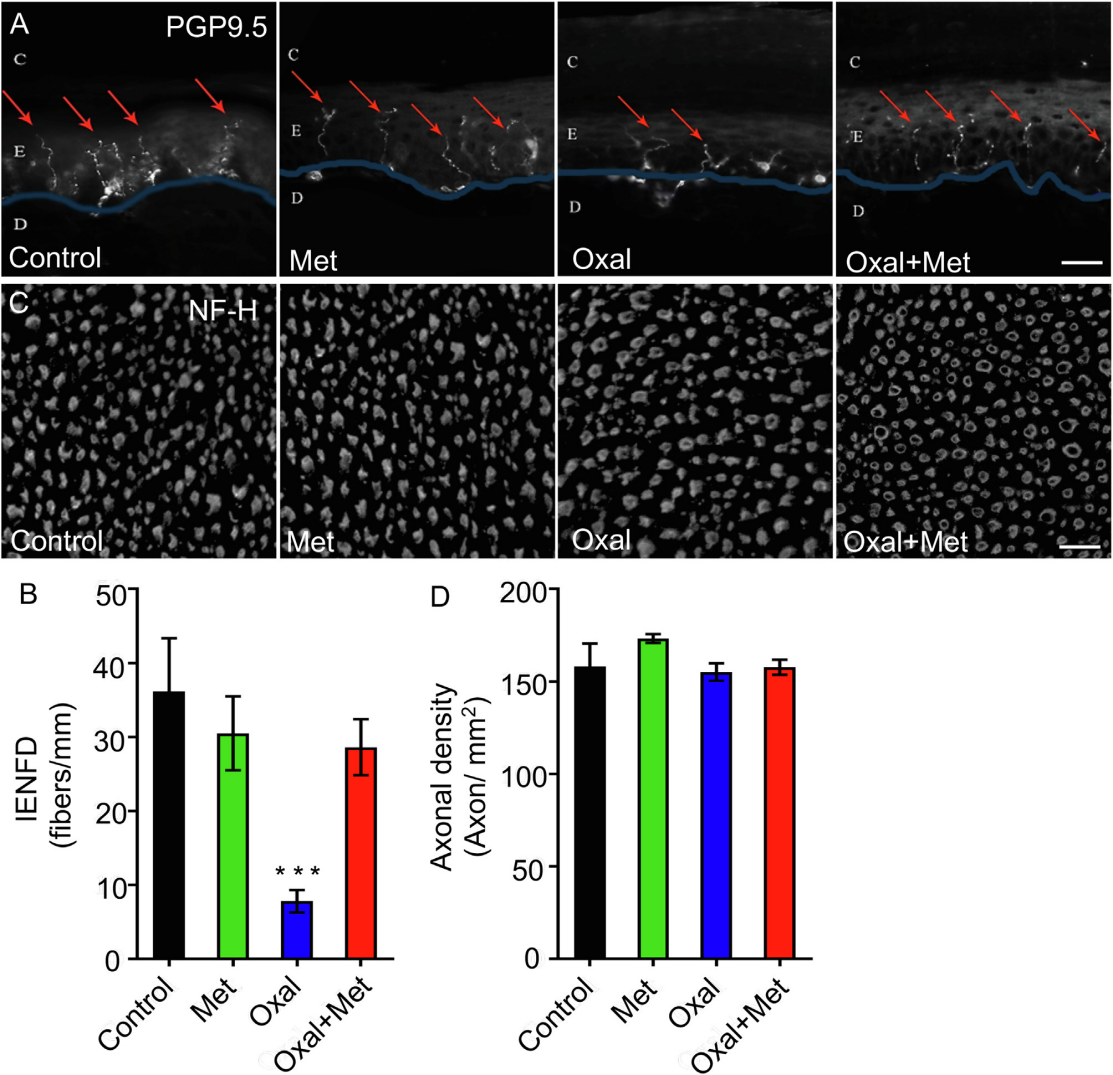
Metformin protects from Oxaliplatin-induced loss of intraepidermal nerve fibers density (IENFD). (A and B) Rats were treated with oxaliplatin (4 mg/Kg i.p. for 2 consecutive days per week), metformin (250 mg/Kg daily i.p.), or both drugs together for 4 weeks. The skin from the paw was removed and immunostained against PGP9.5 (a pan neuronal marker) to quantify the number of nerve fibres crossing into the epidermis per mm (C: *Stratum Corneum*; E: Epidermis; D: Dermis, Scale bar, 20 μm). (Control n = 4; Met n = 10, Oxal n = 10, Oxal + Met n = 10), *** ***p < 0,001 One-Way ANOVA, Bonferroni’s post-hoc test.) (C and D) Sciatic nerves from rats treated with oxaliplatin, metformin, or both drugs were collected, transversally cut and immunostained against anti-neurofilament heavy subunit (NF-H). Axonal density was quantified using epifluorescence reactivity of the neurofilament marker Scale bar, 100 μm- Mean ± SEM, One-Way ANOVA, Bonferroni’s post-hoc test.

As intraepidermal nerve fibre loss has been associated to OIPN (Burakgazi et al., 2011, Han and Smith, 2013), we examined these fibres at the end of the 4 weeks oxaliplatin treatment. Immunostaining with PGP 9.5 was used to visualize the terminations of small-diameter axons within the epidermis of glabrous hind paw skin (Fig. 2A), and intraepidermal nerve fibers density (IENFD) was quantified as detailed in methods (Fig. 2B). Oxaliplatin treatment induced a significant reduction in IENFD, which was completely inhibited by co-treatment with metformin. Importantly, metformin treatment alone did not modified IENFD.

### 2.4 Oxaliplatin did not induce axonal loss at the level of the sciatic nerve

We then examined if axons at the level of the sciatic nerve were affected by oxaliplatin treatment. To this end, transverse sections of sciatic nerves from the different experimental groups were immunostained with anti-neurofilament-H antibody, and axonal density was quantified. We found no differences in axonal density between all experimental groups compared (Fig. 2C and D).

### 2.5 Sensory neurons in the DRG are not affected by oxaliplatin treatment

**Fig. 3:**
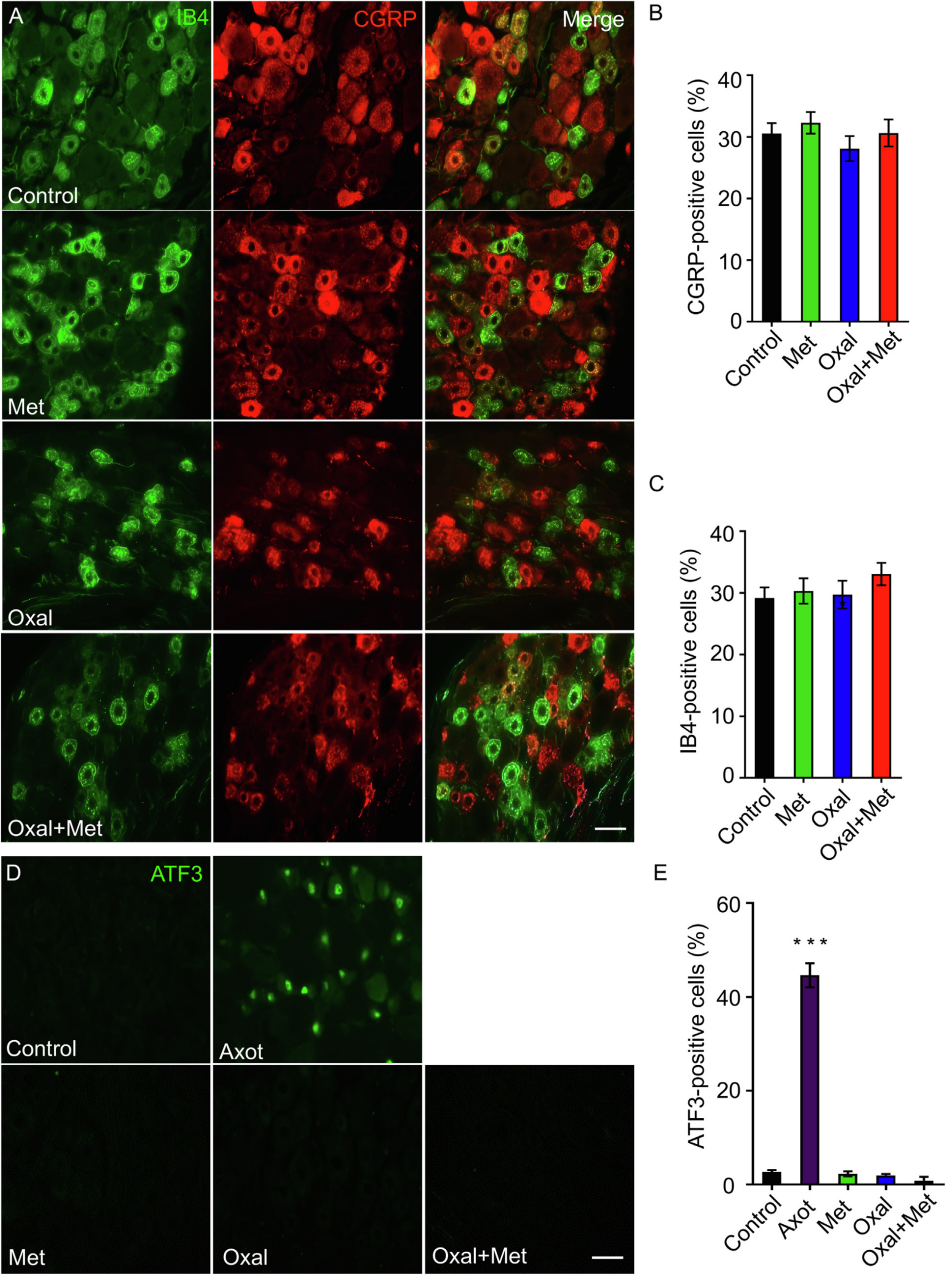
Oxaliplatin did not induce changes in DRG markers (A–C) Images from transverse sections of lumbar sensory dorsal root ganglia from rats after 30 days of treatment with oxaliplatin (4 mg/Kg i.p. for 2 consecutive days per week) and /or metformin (250 mg/Kg daily), labelled with IB4 (non peptidergic neurons) and immunostained against CGRP (peptidergic neurons). Quantification of the percentage of IB4-positive cells and CGRP positive cells are given for the indicated conditions. Scale bar, 20 μm. Mean ± SEM, One-Way ANOVA Bonferroni’s post-hoc test, control n = 3, Met n = 6, Oxal n = 5, Oxal + Met n = 5, 6 sections per animal. (D, E) DRG immunostained for ATF3. We used DRG from axotomized rats as positive control for the immunostaining technique. Quantification of the percentage of ATF3-positive cells in DRGs for the indicated conditions. Control n = 3, Met n = 6, Oxal n = 5, Oxal + Met n = 5, axotomy n = 3; 6 sections per animal. Scale bar, 20 μm. Mean ± SEM, ***p < 0.001, One-Way ANOVA Bonferroni’s post-hoc test.

We next investigated if sub-populations of sensory neurons at the DRG were affected in our OIPN model. To this end, we used staining and antibodies for different populations of lumbar dorsal root ganglia (DRG) neurons. The IB4 lectin binds to the nonpeptidergic population of small-diameter DRG cells, and CGRP is expressed in the majority of peptidergic small diameter DRG neurons (Bennett 2001) and both of these markers have been shown to be downregulated by traumatic nerve injury (Bennett et al., 1998). Nevertheless, in our OIPN model the percentage of DRG neurons binding the lectin IB4 or expressing the neuropeptide CGRP did not change after oxaliplatin treatment, metformin treatment alone, or oxaliplatin plus metformin treatment (Fig. 3A–C).

ATF-3 is a stress-related transcription factor which is up-regulated and translocate to the nuclei after mechanical nerve injuries (Yin et al., 2019). We therefore assessed if a toxic stimulus such as oxaliplatin increases ATF-3 in neuronal nuclei. In control conditions, no immunostaining for ATF3 was detected in DRGs, but sciatic nerve injury dramatically increases ATF-3 positive sensory neurons (used as a positive control) (Fig. 3D and E). Nevertheless, in animals treated with oxaliplatin, metformin or both, no ATF-3 expression was detected (Fig. 3D and E). These results are consistent with the lack of axonal degeneration in the sciatic nerve after oxaliplatin treatment, which nevertheless triggers distal degeneration of thin unmyelinated fibers, an effect completely inhibited by co-treatment with metformin.

## 3 Mild glial activation in the spinal cord induced by oxaliplatin and the effect of metformin

### 3.1 Sensory terminals in the spinal cord are not affected by oxaliplatin treatment

**Fig. 4:**
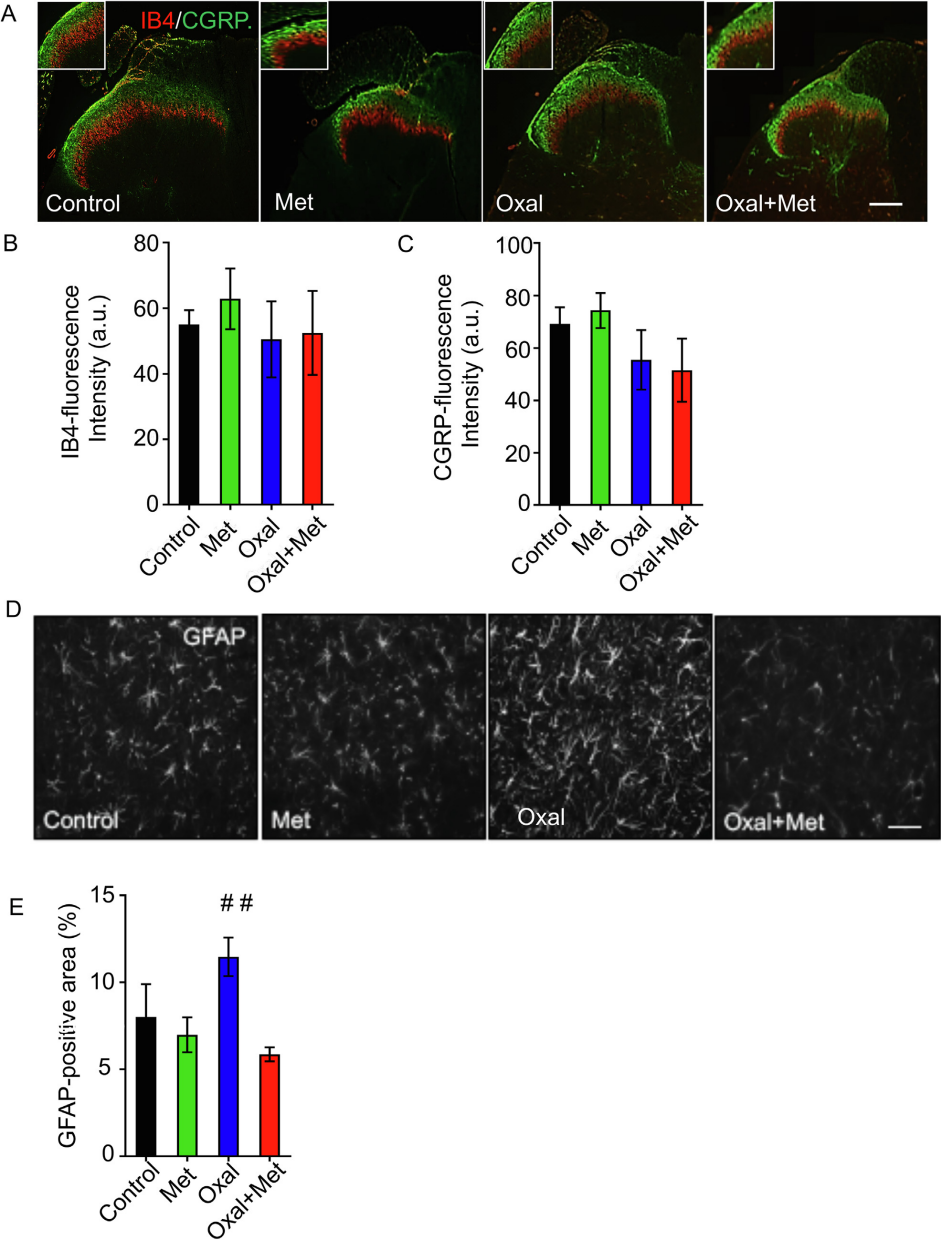
In the spinal cord, oxaliplatin treatment does not change sensory terminals in the dorsal horn, but induces a mild astrogliosis, that can be prevented by metformin. (A) Representative images from transverse sections at the L5 spinal cord level from rats treated for 30 days with oxaliplatin (4 mg/Kg i.p. for 2 consecutive days per week) and/or metformin (250 mg/Kg daily). L5 spinal cord regions were labelled with IB4 (red) and immunostained with CGRP (green) for non-peptidergic and peptidergic neurons, respectively. (B) Quantification of IB4-positive fluorescence intensity for the indicated conditions. Control n = 6, Met n = 6, Oxal n = 5, Oxal + Met n = 5, 6 sections per animal. (C) Quantification of CGRP-positive fluorescence intensity for the indicated conditions. Control n = 6, Met n = 6, Oxal n = 5, Oxal + Met n = 5, 6 sections per animal. One-Way ANOVA Bonferroni’s post-hoc test) (D) Representative images of dorsal horn spinal cord from rats treated for 30 days with oxaliplatin and/or metformin. Sections were immunostained against GFAP to identify astrocytes. Scale bars, 100 μm. (E) Quantification of the percentage of the total area covered by GFAP-positive astrocytes. Mean ± SEM, Control n = 3, Met n = 5, Oxal n = 4, Oxal + Met n = 6, 6 sections per animal. ##p < 0, 01, One-Way ANOVA, Bonferroni’s post-hoc test for comparison between groups. (For interpretation of the references to colour in this figure legend, the reader is referred to the web version of this article.)

It has been previously shown that in addition to degeneration of peripheral terminals in the skin, neurotoxins can induce degeneration of central terminals of sensory neurons located in the dorsal horn of the spinal cord (Huang et al., 2013). Therefore, we assessed changes in markers for sensory terminals approaching the dorsal horn of the spinal cord, at the lumbar segment 5 (L5). In control conditions, IB4-binding nonpeptidergic subpopulation of C-fibres terminates in lamina II–inner (IIi) of the dorsal horn as reported (Snider and McMahon, 1998) and CGRP-positive terminals are found in primary afferent terminations within lamina I and lamina II outer (IIo), as well as less profuse arborisation in deeper laminae (Fig. 4A). Nevertheless, no changes were qualitatively or quantitatively found in the distribution of these sensory terminals after oxaliplatin and/or metformin treatment (Fig. 4A and C).

### 3.2 Astrogliosis induced by oxaliplatin treatment can be prevented by metformin co-administration

As chemotherapy can induce a glial reaction in the spinal cord (Ji et al., 2013, Ruiz-Medina et al., 2013, Robinson et al., 2014), we investigated whether in our OIPN model a similar reaction was taking place. To this end, lumbar spinal cord sections were immunostained with GFAP to label astrocytes, and the area covered by these cells within the superficial dorsal horn was quantified, as glial reaction is associated to increase in glial processes as well as increase in GFAP reactivity in the case of astrocytes (Colburn et al., 1999). Indeed, oxaliplatin leads to an increase in GFAP immunoreactivity, which was completely prevented by metformin co-administration (Fig. 4D and E).

### 3.3 Mechanical and cold hypersensitivity induced by oxaliplatin are prevented by metformin

**Fig. 5:**
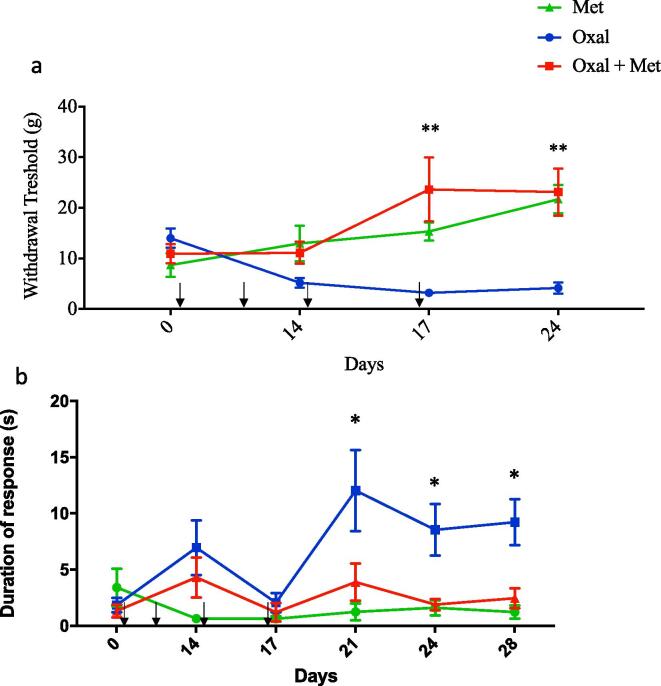
Mechanical and cold hypersensitivity were induced by oxaliplatin, and could be prevented by metformin. Sensory response of rats treated with oxaliplatin (4 mg/Kg i.p. for 2 consecutive days per week) and/or metformin (250 mg/Kg daily) for 30 days. (A) Withdrawal responses to mechanical stimuli (in grams) following the up and down method of Von Frey test. An increased mechanical sensitivity can be observed from the second week onwards compared with control. This hypersensitivity was prevented by metformin treatment (Met n = 7, Oxal n = 12, Oxal + Met n = 7), 2way ANOVA, Bonferronís posthoc test, Mean ± SEM, **p < 0, 01) (B) Acetone test: we measured the duration of pain-like responses following a cold stimuli for the indicated conditions. We observed an increase in the time spent licking or guarding the acetone treated paw (cooled paw) in the oxaliplatin treated animals compared with control. Co administration of Metformin could prevent this effect of oxaliplatin. (Met n = 7, Oxal n = 12, Oxal + Met n = 7), 2way ANOVA, Bonferronís posthoc test, Mean ± SEM, *p < 0, 05).

Having demonstrated a protective effect over pathological changes triggered by metformin, including degeneration of intraepidermal fibres and spinal cord astrogliosis, we performed behavioural tests to evaluate therapeutic effects of metformin over oxaliplatin-induced sensory impairment. Based on the phenotypic traits of the patients suffering from chemotherapy-induced peripheral neuropathy, we evaluated mechanical and cold sensitivity (Saif and Reardon, 2005, Brzezinski, 2012a, Brzezinski, 2012b). To this end, rats were treated with oxaliplatin (4 mg/Kg, i.p.) for two consecutive days per week for four weeks and/or metformin daily (250 mg/Kg, i.p.). Metformin injections started one day before oxaliplatin and were given daily for the duration of the experiment (4 weeks). Compared to metformin alone, oxaliplatin treatment induced a significant decrease in the threshold for mechanical withdrawal responses after the third and fourth cycles, and this was prevented by metformin treatment (Fig. 5A). We then tested the sensitivity to cold stimuli, which commonly increased in patients receiving oxaliplatin. For this, we quantified the time the animal spent licking, guarding or shaking the paw after acetone application, which produce a localized cold stimulus. Oxaliplatin treatment increases the time rats spend presenting pain like responses, especially after 3 cycles of oxaliplatin, when doses have accumulated. This response that was completely inhibited by co-treatment with metformin (Fig. 5B).

## 4 Discussion

In this study we show that metformin, a widely used antidiabetic drug, protects axonal degeneration *in vitro* and in a rat model of oxaliplatin induced neuropathy. Oxaliplatin induced degeneration of axons in explants of dorsal root ganglia, and this could be prevented by metformin treatment. Rats exposed to oxaliplatin developed degeneration of intraepidermal fibers, activation of astrocytes in the spinal cord and mechanical and cold hyperalgesia, all of which were prevented by metformin co-administration. Taken together, this data strongly suggests that metformin could reduce the incidence of OIPN in cancer patients.

It has been previously demonstrated that oxaliplatin, and other drugs used as chemotherapeutics, induces axonal degeneration of *in vitro* sensory neurons (Court and Coleman, 2012, Lopez-Gonzalez et al., 2018). In this study we first confirmed this observation by treating DRG explants with oxaliplatin and demonstrate that metformin strongly delays oxaliplatin-induced axonal degeneration. Axonal degeneration is associated to calcium release from the ER, activation of the mitochondrial permeability transition pore (mPTP) and ROS production (Barrientos et al., 2011, Villegas et al., 2014). More recently, we have shown that axonal degeneration is dependent on the activation of the necroptotic machinery (Arrazola et al., 2019). It has been demonstrated that oxaliplatin can trigger increase in ROS, as well as mitochondrial dysfunction (Canta et al., 2015), processes associated to necroptosis activation, and metformin can inhibit both processes. Therefore, it will be interesting in the future to establish the mechanisms associated to axonal degeneration mediated by oxaliplatin and the protection conferred by metformin, to generate novel therapeutic interventions.

We next moved to an *in vivo* model of oxaliplatin induced neuropathy. A similar rat model of oxaliplatin induced neuropathy has been published (Boyette-Davis and Dougherty, 2011), which is similar to the model we used although the dose was smaller and given for less days. We ought to increase the dosing in order to achieve a clinically relevant model of OIPN (Miyagi et al., 2019). An important consequence of oxaliplatin treatment was the degeneration of intraepidermal fibres, which has been reported in other painful neuropathies (Lauria et al., 2009), including ddC- and D4T-induced neuropathies (Huang et al., 2013, Huang et al., 2017), HIV-mediated neurotoxicity (Wallace et al., 2007), and paclitaxel-induced neuropathy (Lauria et al., 2005, Bennett et al., 2011). We explored axons at the level of the sciatic nerve and observed no changes in fibre density after oxaliplatin treatment. This observation is in agreement with results after anti-retroviral treatments (Huang et al., 2013, Huang et al., 2017), as well as in paclitaxel and epothilone-B induced neuropathies (Flatters and Bennett, 2006, Carozzi et al., 2010), but differs to what has been seen in diabetic neuropathy, where an increase in axonal density in the sciatic nerve has been reported (De Gregorio et al., 2018). This discrepancy may be explained by the longer duration of the disease in diabetic animals or by the different mechanism and intensity of the injury. We did not detect DRG abnormalities after oxaliplatin treatment, which is in contrast to models of nerve injury. ATF3 was not increased, nor was there any change in the density of CGRP, and IB4 in small- and large-diameter DRG neurons, which has been observed in nerve injury-induced neuropathy models (Gehrmann et al., 1991, Hu and McLachlan, 2002, Peters et al., 2007), but not in chemotherapy induced neuropathies (Flatters and Bennett, 2006). Furthermore, we did not observe changes in the central projections of primary afferents in the spinal cord, as has been reported in anti-retroviral induced neuropathy (Huang et al., 2013). In contrast to an early microgliosis followed by astrogliosis in the dorsal horn after nerve injury (Beggs and Salter, 2007, Calvo and Bennett, 2012), we only found a small increase in astrogliosis. Indeed, it has been reported that other chemotherapeutic agents that cause neuropathic pain and intraepidermal fibre degeneration, increase glial activation in the spinal cord (Zheng et al., 2011). This is relevant as it is now clear that neurotoxic ‘dying back’ neuropathies are distinct from those evoked by nerve injury models, emphasizing the need to develop animal models relevant to specific clinical scenarios. Similar to previous reports, we observed mechanical allodynia and cold hyperalgesia in oxaliplatin treated rats, which could be explained by the pathological changes described above.

Metformin is a widely used anti-diabetic drug that activates adenosine monophosphate-activated protein kinase (AMPK) (Rena et al., 2017). Metformin has antioxidant properties through its ability to reduce reactive oxygen species (ROS), nitric oxide (NO), and other oxidative stress markers (Hou et al., 2010). Also, metformin reduces ROS production by diminishing the activity of the mitochondrial respiratory chain (Ouslimani et al., 2005). Recent findings on animal models showed that metformin reduces neuropathic pain through AMPK activation and inhibition of mammalian target of rapamycin (mTOR) (Melemedjian et al., 2011, Tillu et al., 2012, Melemedjian et al., 2013, Zhang et al., 2013). In addition, it has been recently demonstrated that activation of AMPK inhibits necroptosis (Lee et al., 2019), a cell death mechanism associated to several neurodegenerative conditions and involved in axonal degeneration after diverse stimuli (Zhang et al., 2017, Hernandez et al., 2018, Arrazola et al., 2019). On the other hand, oxaliplatin induces enhanced synaptic plasticity by decreasing AMPKα activity and thus metformin and its effect on inducing the mTOR/p70S6K signal pathway seems a plausible mechanism of action (Ling et al., 2017).

Metformin co-administered to oxaliplatin treated rats prevents the pathological and functional changes seen in oxaliplatin-treated animals. One caveat of this study is that we only used male rats, and we know that chronic pain may have gender differences (Greenspan, 2007). However, we could only have access to male rats. Ideally, this study should be repeated in female rats. These results are consistent with the observation of a protective effect of metformin in cisplatin-induced peripheral neuropathy in a mouse model (Mao-Ying et al., 2014). This gives a new avenue of prevention for the development of chemotherapy induced neuropathy. Interestingly, in a recent small RCT, metformin 500 mg given three times daily to oxaliplatin treated-stage III colorectal cancer patients was shown to reduce the incidence of neuropathy and the intensity of pain (El-Fatatry et al., 2018).

In summary, our results show that metformin is effective to prevent oxaliplatin-induced neuropathy in a rat model, providing mechanistic evidence to evaluate this approach in a large clinical trial.

## 5 Materials and methods

### 5.1 Animals

Adult Sprague Dawley male rats weighting 250 g at the beginning of the experiment were purchased from Harlan Biosciences. Rats were housed on a 12 h light/dark cycle with ad libitum food and water. Experiments with animals followed protocols approved by the Institutional Animal Care and Use Committees and complied with National Institutes of Health guidelines. We report this study in compliance with the ARRIVE guidelines 63 (20 points checklist).

### 5.2 In vitro determination of axonal degeneration

E16 rat embryos were decapitated, and the vertebral column was removed. Spinal cord with dorsal root ganglia (DRG) were dissected and placed in a Petri dish containing L-15 medium. For DRG explants, complete DRGs were cultured in 24-well dishes containing 400 mL of Neurobasal medium (Invitrogen), 2% B27 (Invitrogen), 0.3% L-glutamine, 1% streptomycin/penicillin, 4 mM aphidicolin, 7.5 g/ml 5-fluoro-2- deoxyuridine, and 50 ng/ml NGF. DRGs were cultured for 7d at 37 °C and 5% CO2. At day 7, ganglia were treated with 10 *u*M Oxaliplatin and/or 10 mM Metformin. The percentage of axonal degeneration was based on the ratio of the areas of fragmented axons versus total axonal area (Villegas et al., 2014). Degenerated axon fragments were detected using the particle analyzer algorithm of NIH ImageJ, and the total fragmented axon area versus total axonal area was used to estimate the axonal degeneration.

### 5.3 Oxaliplatin and metformin treatment

We used adult Sprague Dawley male rats (250 g) that were injected intraperitoneally (i.p.) with Oxaliplatin (Oxaliplatin, Ebewe Recalcine, 4 mg/Kg i.p.) in two consecutive days every week, for 4 weeks. Rats were randomly assigned (by a computer generated sequence) to one of each group Metformin alone (Met; n = 15), Oxaliplatin alone (Oxal; n = 15) and cotreatment of Oxaliplatin and Metformin (Oxal + Met; n = 15). Metformin (250 mg/kg) was administrated daily by intraperitoneal injections for 4 weeks. Oxaliplatin (4 mg/Kg) was administered intraperitoneally two consecutive days per week during four weeks. Metformin injections were administered 6 h before Oxaliplatin injections in the days that both drugs were used in the Oxal + Met group. Glycaemia was determinate using a Code Free blood glucose monitoring system (SD) and haematological toxicity was evaluated collecting blood directly from the tail snip.

### 5.4 Histology

After a defined survival time, animals were terminally anaesthetized with Ketamine/Xilazin (1:1) and transcardially perfused with 4% paraformaldehyde (PFA) in 0.1 M phosphate buffer (PB). We collected glabrous plantar skin, L4 and L5 DRG, and the sciatic nerves. Tissue for immunohistochemistry was post-fixed in 4% PFA for 2 h, followed by three 10 min washes in PBS, sucrose gradient (5%, 10%, 20% in PBS), and then embedded in OCT (Sakura Finetek).

Samples were sectioned at 14 μm (skin), 8 μm (nerves and DRGs) and 10 μm (spinal cords) and mounted on Superfrost Plus slides (Thermo Fisher Scientific). Sections were washed in 1x PBS for 10 min and then blocked/permeabilized in 0.1% Triton X-100, 2% fish skin gelatin (Sigma-Aldrich) in 1X PBS for 1 h at RT. Sections were incubated in primary antibodies in blocking/permeabilizing solution overnight at 4 °C, washed in 1XPBS 3x10 min, and incubated in secondary antibodies for 2 h at RT. Sections were washed 3x10 min in 1XPBS and mounted in Vectashield (Vector Laboratories). See Supplementary Table 1 for antibodies used.

### 5.5 Quantification and analysis of IF

Intraepidermal fibres from skin biopsies were counted live on the microscope at a 40X magnification as previously described (Lauria et al., 2005). Only single fibres crossing the dermal–epidermal junction were counted, excluding secondary branching from quantification. The length of the section was measured using Image J and used to calculate the epidermal innervation density (IENF/mm). Quantification of axons in the sciatic nerve was done quantifying epifluorescence reactivity of the neurofilament marker (matched for laser power, photomultiplier tube gain/offset, and post processing) by thresholding and binarization using the particle analysis macro of the open source ImageJ software. For IB4, CGRP, or ATF3 expression in the DRG analysis was performed from 6 randomly selected sections from each animal; the total number of DRG cell profiles and the number of profiles expressing immunoreactivity for the protein of interest was counted and reported as percent over the total. Cell profiles were sampled only if the nucleus was visible within the plane of section and if cell profiles exhibited distinctly delineated borders. GFAP in the spinal cord was measured in 6 randomly selected L5 spinal cord section from each animal. IB4 and CGRP in the dorsal horn were also analysed measuring the intensity of immunoreactivity in laminae I and II and expressed in arbitrary units (AU). ROIs were defined using the demarcation tool in ImageJ to encircle lamina I and II Particle analysis was performed on equalized images. Equalization was reached as a result of equivalent histological processing and identical image acquisition parameters between samples. After, background intensity threshold was determined on control group images and then used on the analysis of all groups. Intensity values were normalized by the total area quantified. All the studies were analyzed by a blinded single observer.

### 5.6 Behavioural tests

Tests were performed on male rats of the three different treatment groups. Mechanical allodynia was assessed as the hind paw threshold withdrawal response to Von Frey filament touch. Rats were placed in a transparent box over a mesh floor. There, stimulation was done following the up and down method (Calcutt et al., 1996). Von Frey filaments were applied perpendicular to the mid-plantar area of right hind paw starting with the 5.18 g filament. A positive response was defined as a paw withdrawal or shaking after 2 s touch. After any positive response, the next lower filament was applied, and in case of a negative response the next higher filament was applied. The testing session consist in 5 trials after the first change in response. The sequence of response was converted into 50% withdrawal threshold using the formula: 50% PWT = 10^(X+kδ)^_f_ /10^4^, where X_f_ is the value of the final von Frey filament used (in log units), k is a value measured from the pattern of positive/negative responses, and *δ* = 0.21, which is the average interval (in log units) between the von Frey filaments. (Calcutt et al., 1996). Three sessions of habituation (30 min each) to the testing area were conducted in three consecutive days followed by two baseline measurements of hind paw withdrawal threshold in another two consecutive days. To measure cold allodynia, we applied a drop of acetone to the plantar hind paw and measured the time that the animal spent licking, shaking, guarding or lifting the paw during the following 2 min (Kontinen and Dickenson, 2000). Baseline values were taken three times before the first drug administration.

### 5.7 Statistical analysis

For histological analysis, data sets were tested for normality by the Kolmogorov–Smirnov test and for homogeneity of variance by Levene’s test. Parametric or nonparametric tests were used accordingly. Behavioral data were analysed using two-way ANOVA. Data are reported as mean values ± standard error of the mean. P < 0.05 was considered as statistically significant. All statistics were done using *GraphpadPrism6* software.

## CRediT authorship contribution statement

**N.W. Martinez:** Investigation, Methodology, Formal analysis, Writing - original draft, Visualization. **A. Sánchez:** Investigation, Methodology. **P. Diaz:** Investigation, Methodology. **R. Broekhuizen:** Investigation, Methodology. **J. Godoy:** Visualization. **S. Mondaca:** Visualization. **A. Catenaccio:** Investigation, Methodology. **P. Macanas:** Project administration. **B. Nervi:** Supervision, Conceptualization. **M. Calvo:** Supervision, Conceptualization, Writing - review & editing, Investigation, Methodology, Formal analysis. **F.A. Court:** Supervision, Conceptualization, Writing - review & editing.

## Declaration of Competing Interest

The authors declare that they have no known competing financial interests or personal relationships that could have appeared to influence the work reported in this paper.
