# Supplementary material for: Metformin protects from oxaliplatin induced peripheral neuropathy in rats
Source: Neurobiol Pain. 2020 May 22;8:100048. doi: 10.1016/j.ynpai.2020.100048 (PMC7260677; doi:10.1016/j.ynpai.2020.100048)
Supplement: Supplementary data 1 [file mmc1.docx]

**Table S1. Primary Antibodies Used.**

| **Species Reactivity** | **Antibody** | **Supplier** | **Catalogue Number** | **Host** | **Dilution** |
| --- | --- | --- | --- | --- | --- |
| Rat | Anti-Neurofilament Heavy Chain | Sigma-Aldrich | #N4142 | Rabbit | 1:1000 |
| Rat | Anti- Acetylated Tubulin | Sigma-Aldrich | #T6793 | Mouse | 1:1000 |
| Rat | Anti -PGP 9.5 | Ultraclone | Limited #31A | Rabbit | 1:1000 |
| Rat | Anti-Glial Fibrillary Acidic Protein (GFAP) | Dako | #N1506 | Rabbit | 1:1000 |
| Rat | Anti-Calcitonin Gene Related Peptide (CGRP) | Sigma-Aldrich | #C8198 | Rabbit | 1:1000 |
| Rat | Anti- Activating Transcription Factor (ATF3) | abcam | Ab87213 | Rabbit | 1:1000 |

**Table S2. Secondary antibodies used.**

| **Antibody** | **Supplier** | **Catalogue Number** | **Host** | **Immunofluorescence**  **Dilution** |
| --- | --- | --- | --- | --- |
| anti-Mouse Alexa 488 | Molecular Probes | #A11029 | Goat | 1:1000 |
| Anti-Rabbit Alexa 546 | Molecular Probes | #A11035 | Goat | 1:1000 |

**Table S3. Markers used.**

| **Marker** | **Supplier** | **Catalogue Number** | **Immunofluorescence**  **Dilution** |
| --- | --- | --- | --- |
| Lectin from Griffonia simplicifolia (IB4) | Sigma-Aldrich | L2140 | 1:50 |
| ExtrAvidin-FITC | Sigma-Aldrich | #E2761 | 1:500 |

**Supplementary Figure Legends**

**Supplementary Figure 1. Oxaliplatin and/or metformin have no overt effect over glycaemia, weight gain and white blood cell counting.** Glycaemia, weight gain and white blood cells counting of rats treated during 30 days of treatment with oxaliplatin (4mg/Kg) and/or metformin (250mg/Kg). **(A)** Blood sugar measurements for the indicated treatments. Glycaemia was determined by analysis of blood samples extracted from the tails into a blood glucose monitor at days 0, 14, 19 and 29 of this study (n=3 in all groups) **(B)** Weight gain percentage quantification for the indicated treatments. Weight of all rats ranged between 200g and 250g at the beginning of this study. Weight gain percentage was determined by normalizing data with the previous time point at days 0, 15, 22 and 29 of this study. Oxaliplatin and cotreatment induced a significant delay in weight gain at day 22. However, all the groups registered a sustained increase in weight gain. . (Met n=8, Oxal n= 15, Oxal + Met n= 9) **(C)** White blood cell concentration for the indicated treatments. White blood cells levels were determined by analysis in Haemocytometer of blood samples extracted from the tails at days 0, 14, 19 and 29 of this study. (n=3 in all groups)

Mean ± SEM, ** indicates differences (p<0, 01) and **** (p<0, 0001) for oxaliplatin treatment or cotreatment of oxaliplatin and metformin compared to metformin treatment, using Two-Way ANOVA. Bonferroni’s post test was used for comparison between groups.
