# Supplementary figures and images for: Metformin protects from oxaliplatin induced peripheral neuropathy in rats
Source: Neurobiol Pain. 2020 May 22;8:100048. doi: 10.1016/j.ynpai.2020.100048 (PMC7260677; doi:10.1016/j.ynpai.2020.100048)

Supplementary figure 1

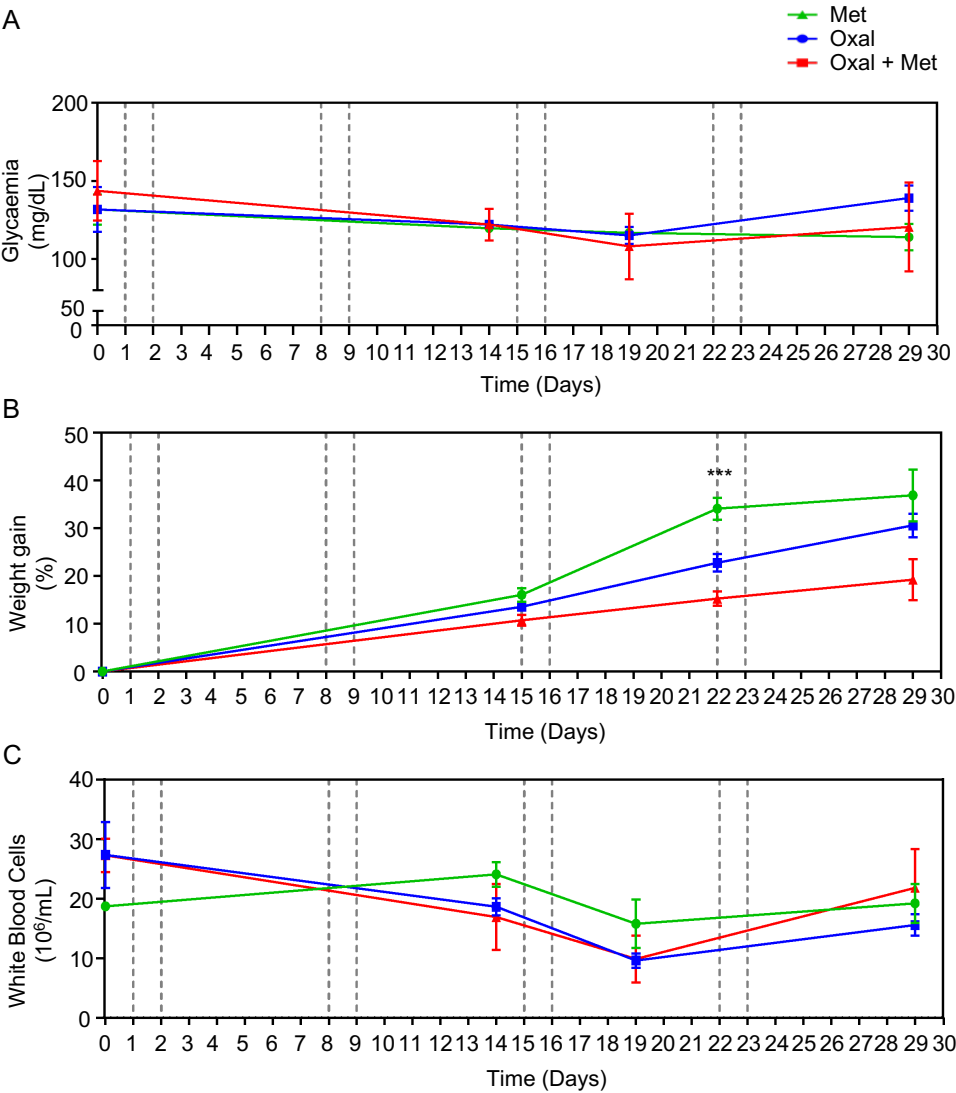

Supplement: Supplementary data 2 [file mmc2.pdf]
